# The Relationship between Free Volume and Cooperative Rearrangement: From the Temperature-Dependent Neutron Total Scattering Experiment of Polystyrene

**DOI:** 10.3390/polym13183042

**Published:** 2021-09-09

**Authors:** Zehua Han, Guisheng Jiao, Changli Ma, Taisen Zuo, Charles C. Han, He Cheng

**Affiliations:** 1 Spallation Neutron Source Science Center, Dongguan 523808, China; hanzh@ihep.ac.cn (Z.H.); machangli@ihep.ac.cn (C.M.); 2 Institute of High Energy Physics, Chinese Academy of Sciences, Beijing 100049, China; 3 Department of Physics, University of Chinese Academy of Sciences, Beijing 100049, China; 4 Shenzhen Key Laboratory of Polymer Science and Technology, Guangdong Research Center for Interfacial Engineering of Functional Materials, College of Materials Science and Engineering, Shenzhen University, Shenzhen 518055, China; jiaogs@szu.edu.cn; 5 College of Physics and Optoelectronic Engineering, Shenzhen University, Shenzhen 518060, China; 6 Institute for Advanced Study, Shenzhen University, Shenzhen 508060, China; han.polymer02@yahoo.com

**Keywords:** glass formation, neutron scattering, molecular dynamic simulation, free volume, cooperative rearrangement

## Abstract

Although many theories have been proposed to describe the nature of glass formation, its microscopic picture is still missing. Here, by a combination of neutron scattering and molecular dynamics simulation, we present the temperature-dependent atomic structure variation of polystyrene at the glass formation, free volume and cooperative rearrangement. When it is close to glass formation, the polymer is confined in tubes, whose diameter is the main chain–main chain distance, in a “static cage” from its neighbors. This definition can not only account for the kinetic pathway dependence of Williams-Landel-Ferry (WLF) free volume, but also be testified in a set of six polymers. However, the free volume which allows a monomer to move cannot be found in any frame of its real-space image. Monomers, thus, have to move cooperatively to be out of the cage. During glass formation, dynamic heterogeneity develops, and string-like cooperative rearrangement region (CRR) grows over a long range of time and length scales. All of these CRRs tend to walk through loose “static cages”. Our observation unifies the concepts of free volume and cooperative rearrangement. The former is a statistical average leading to a polydisperse “static cage” formation; while a loose “static cage” provides the way that CRRs move.

## 1. Introduction

Vogel first proposed the concept of free volume in 1921 [1]. In theory, it looks easy to understand, i.e., a polymer can move only when it has the space to do so. However, the free volume in a polymer melt has never been measured directly, and different models have to be chosen to estimate the occupied volume [2,3,4,5,6]. Before comparing the free volumes from different groups, their distinct definitions have to be clarified. The relationship between the “void” measured by those techniques (such as the positron annihilation lifetime spectroscopy, gas absorption and birefringence measurements) and free volume is hard to judge [7]. In scattering methods, the only experimental observation which may be related to the molecular packing density and possibly the “free volume” has been the small change in the scattering function, S(*q*), at the scattering vector (*q*) in the range of the monomer dimension [8]; while in the simulation, some groups have tried to use thermodynamic approaches to calculate the free volume [9,10,11,12] As a result, the William-Landau-Ferry (WLF) theory is often believed to be phenomenological [13,14]. 

In contrast to this free volume approach, Adam and Gibbs proposed that the polymer could still move if they performed this in a cooperative way at a low temperature [15]. Their starting physical idea is that the relaxation dynamics at low temperature are the result of a sequence of individual events in which a subregion of the system relaxes to a new local configuration [8]. Confocal microscopy can be used to directly track 2D and 3D dynamics of colloidal particles in supercooled fluids [16]. Cooperative rearrangement regions (CRRs) and heterogeneous dynamics are observed in both repulsive and attractive glasses. CRRs are observed to be string-like in repulsive glass and compact structures in attractive glass [17].

Therefore, the microscopic picture of glass dynamics is very important to clarify the problem. Richet P. et al. summarized the nature and history of glass in the recent Encyclopedia [18]. Additionally, scientists have tried almost all methods to study glass dynamics. For small molecules, such as solvents and metal alloys, the Pair Distribution Function (PDF) analysis with X-ray or neutron diffraction can reveal the atomic structural changes. However, it cannot be used in an amorphous polymer field because of the limitation of the observation range [19]. The microscopic image of glass formation in the polymer field is still missing. Neither real nor reciprocal space observation methods can directly see the monomer movement. The observation method in real space, such as the confocal microscope, has a resolution hundreds of times larger than the size of monomers; thus, the monomer cannot be seen. The reciprocal observation method, such as X-ray photon correlation spectroscopy, is actually used to observe probe motion, while the size of the probe is also hundreds of times larger than that of the monomer, and adding the probe would affect the dynamics [20]. As a result, we still do not have an intuitive image of free volume, and we do not know whether polymers rely on free volume or CRR motion during glass formation either.

Developments in Neutron Total Scattering techniques facilitate the continuous structural measurements covering the length scale from 0.01 angstrom to 10 nanometers. Based on the fact that the deuterated polymer has the same atomic structure [21], but a different neutron contrast with its hydrogenate counterpart, the new instrumentations NIMROD and NOVA when combined with the deuterium labelling technique and molecular dynamics (MD) simulation allows us to visualize the most probable all-atom positions in a disordered polymer. In the previous study, we carried out a series of neutron total scattering measurements (three Polystyrene (PS) homopolymers), with the same molecular weight and molecular weight distribution, i.e., perdeuterated PS-d8, phenyl deuterated PS-d5, hydrogenous PS-h8 and three of their binary blends) and corresponding MD simulations at different temperatures during glass formation. When the Fourier transforms of the real space MD simulation are in agreements with all of those scattering profiles at different temperatures, as well as the neutron scattering of backbone-deuterated PS-d3 and X-ray results in the literature [22,23], the real space MD images represent the most probable all-atom positions in PS [24]. 

In this manuscript, we further assume MD images can be regarded as frames of a film, demonstrating both the statics and dynamics of glass formation at different length and time scales. The temperature dependence of free volume and cooperative rearrangement can be revealed experimentally. The manuscript consists of three parts. In the first part, we obtain a “static cage” structure from the neutron profiles of PS and propose an equation to predict fractional free volume in the WLF equation, while in the second part, coarse-graining simulations are conducted at longer length and time scales at different temperatures based on the force fields of the all-atom simulation. Then, general microscopic images with CRRs and dynamic heterogeneity can be seen without monomer details. Finally, a microscopic picture of glass formation is posed. We believe that dynamic slowing down induces glass formation. Free volume, CRRs, dynamic heterogeneity and the α/β split can all be linked by it. In polymer melts at temperatures away from glass formation, thermal fluctuation enables neighbor segments around a polymer chain to move freely. The decrease in temperature lowers the amplitude of thermal fluctuation, leading to the decrease in free volume. As a consequence, both the static and dynamic cages form; polymer chains are confined in tubes whose average diameter is the main chain–main chain distance at glass formation in the “static cage”, although the molecular weight of PS in this study is much lower than its entanglement molecular weight. The fractional free volume is defined by the volume ratio of the space outside of the tube over the “static cage” size. Here, the fractional free volume which consists of the WLF equation is a statistical average, and the numbers of the monomer in the “static cage” are polydisperse, i.e., some of the “static cages” are crowded and some of them are loose. However, the spherical free volume which enables the monomer to move cannot be found in any frame of real-space images. Monomers, thus, have to move cooperatively, such as a string, to come out of the cage. Dynamic heterogeneity develops over a long range of time and length scales. Additionally, faster strings always move in loose “static cages”.

## 2. Materials and Methods

### 2.1. Material

Samples for total scattering were prepared by classic anionic polymerization of styrene and deuterated styrene purchased from Sigma-Aldrich (Shanghai, China) and Reer Technology Ltd (Shanghai, China) with *sec*-butyllithium as the initiator in benzene at 25 °C [24]. Three homopolymers, PS-d8 (totally deuterated), PS-d5 (phenyl deuterated) and PS-h8 (hydrogenated), with almost the same molecular weight (M_w_ ~ 8900 g/mol) and molecular weight distribution (M_w_/M_n_ ~ 1.1), were synthesized.

### 2.2. Sample Preparing and Neutron Total Scattering

Neutron total scattering experiments were carried out on the NIMROD diffractometer at the ISIS Pulsed Neutron Source (STFC Rutherford/Appleton Laboratory, Didcot, UK) and the NOVA diffractometer at the Materials and Life Science Experimental Facility (MLF) in J-PARC (Tokai, Japan) [25,26]. A simultaneous scattering vector (Q) range of 0.02–50 Å^−1^ was achieved. The scattering measurements were performed on 24 × 24 × 1 mm^3^ sample in null scattering Ti/Zr flat plate cells or Aluminum cells with about 1 mm thick window. The samples were heated and monitored by two heaters and two thermometers. The sample temperature was first kept at 453 K for 20 min to eliminate thermal history. Then, it was deceased to the measurement temperatures at a cooling rates of 1 K/min. Neutron total scattering was conducted at 453 K, 438 K, 423 K, 405 K, 393 K, 358 K, 343 K and 328 K, respectively. The measurement time of each sample at each temperature was from 2 to 4 h depending on the content of hydrogen. Because of the low cooling rate, there should have been no hysteresis result [27]. Empty cell backgrounds and a 3 mm thick vanadium plate calibration standard were also measured for an equivalent amount of time. Each raw scattering data were corrected for instrument and sample holder backgrounds, attenuation and multiple scattering using the instrument specific software Gudrun [28]. The reduced scatterings were then normalized against the known scattering of the vanadium calibration standard and converted to the microscopic differential scattering cross-section ∂σ(Q)/∂Ω vs. Q for total scattering analysis. 

### 2.3. Molecular Dynamics Simulations

The all-atom simulation (AA simulation) system contains 47 PS chains of length 88 at different temperatures as our experiments. Fully atomistic simulations were carried out by the software package Gromacs–2016.5 under isothermal–isobaric (NPT) conditions at 1 bar using the Nosé-Hoover thermostat (coupling time 0.2 ps) and Parrinello–Rahman barostat (coupling time 1.0 ps) [29]. An integration time step of 1 *fs* was used. The nonbonded interaction cutoff was *r_c_* = 1.0 nm. Additionally, the force field of PS was the all-atom Optimized Potential for Liquid Simulation (OPLS-AA) [30,31,32]. More details can be found in our previous work [18].

The coarse-grained (CG) model was conducted by the structure-based iterative Boltzmann inversion (IBI) method 26. This method assumes the total potential of the system U^CG^ consists of two parts, a bonded (U^CG^-bonded) and a nonbonded (U^CG^-nonbonded) part. The bonded potentials were approximated by the potentials of mean force of CG degrees of freedom (bond lengths (*r*), angles (*θ*) and dihedral torsions (*φ*)). The independent bonded potentials, assuming these potentials are uncorrelated, are usually given by simple Boltzmann inversion:

(1) $$U^{CG}(r,\theta ,\varphi )=-k_{B}T\ln P^{CG}(r,\theta ,\varphi )$$

where the *P^CG^*(*r*, *θ*, *φ*) is the distribution function of bond length (*r*), bond angles (*θ*) and torsion angles (*φ*). For the nonbonded interaction between the CG beads, the potential of mean force can be used as a first guess in an iterative refinement:

(2) $$U_{0}^{CG}(r)=-k_{B}T\ln g^{target}(r)$$

where *g^target^*(*r*) is the target radial distribution function (RDF) from the reference atomistic simulation. Then, modifying the potential according to the difference between the calculated and target RDFs is iterated in the following way until the two RDFs match:

(3) $$U_{i+1}^{CG}(r)=U_{i}^{CG}(r)+k_{B}T\ln(g_{i}^{calculated}(r)/g^{target}(r))$$

where $g_{i}^{calculated}(r)$ is the RDF calculated with the potential and $U_{i}^{CG}(r)$ in the *i*th iteration. At last, to correct the pressure, a linear perturbation was added to the potential:

(4) $$\Delta U_{lin}(r)=A(1-\frac{r}{r_{cut}})$$

where *A* is a small constant. This linear modification of the potential and the structure-based iterations of Equation (3) were performed concurrently until the target pressure was obtained. More details can be found in Ref. [26].

In our coarse-grained model, one CG bead represented one PS monomer and it was centered on the corresponding centers of mass. 

All CG simulations in this work were carried out by HOOMD-blue package [33,34]. The Nosé-Hoover thermostat (coupling time 0.18 ps) was used. The nonbonded interactions were truncated beyond 1.3 nm with a neighbor list cut-off of 1.4 nm. The time step was set up to 5 fs. Here, 453 K was set as T = 1.0. Additionally, glass formed when T = 0.4, its corresponding real temperature was 181.2 K. The cooling rate for CG simulation was 0.02 K/ps. 

There are three T_g_ in the manuscript. The first T_g_ was measured by neutron total scattering from the temperature-dependent iso-thermal expansion factor; the other two in AA and CG simulations were from the inflection point of the temperature-dependent density curves. The T_g_ in neutron scattering was the same with that in AA simulation (it was also the same with DSC measurement). However, the T_g_ in CG simulation was much lower than the other two. The reason can be attributed to the construction method of CG model. It is a structure-based methodology without the dynamic correction. The loss of degrees of freedom will accelerate the dynamic behavior of the system, then the T_g_ will decrease in CG model in comparison with the AA model. Because it is still a reliable way to extend the length and time scales, and widely used in the simulations in polymer field [35], we investigated the dynamics of the system by CG simulation.

### 2.4. Fourier Transforms of MD Simulations

**Figure 1:**
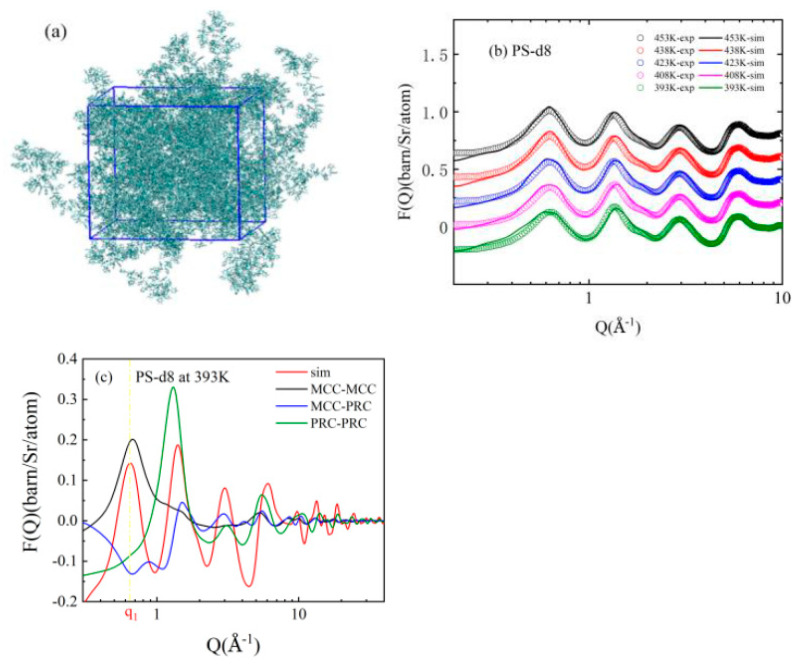
Neutron total scattering and all-atom simulation; (**a**) 3D atomic structure of PS at 393 K; (**b**) neutron total scattering profiles of PS-d8 at different temperatures and the Fourier transform from (**a**); (**c**) the decomposition of *q*_1_ peak of PS-d8 at 393K, the yellow dash lines were used to guide the eye.

Neutron total scattering profiles of six samples (PS-d8, PS-d5, PS-h8, 50 mol% PS-d8/50 mol% PS-h8, 50 mol% PS-d8/50 mol% PS-d5 and 50 mol% PS-d5/50 mol% PS-h8) as shown in Figure 1b and Figure S1 were compared with the Fourier Transforms of molecular dynamics simulations. The sizes of the simulation boxes were about 90 Å. According to the Periodic Boundary Conditions (PBCs), the smallest accessible scattering vector was about 2π/90 = 0.07 Å^−1^. Scattering profiles with scattering vector lower than 0.07 Å^−1^ had to be calculated without the PBCs. To keep us in a safe condition, all of the scattering curves with scattering vector from 0.3 Å^−1^ to 40 Å^−1^ (we call it diffraction curves) were calculated with the PBCs. 

### 2.5. Define Cooperative Rearrangement Regions

There were 2 steps to categorize the CRR in a polymer system. 

First, choose the 10% fast monomers. The time interval was when χ_4_ reached its maximum. During this time interval, the mean squared displacement (MSD) of each monomer could be derived. Then, we chose those monomers with the 10% largest MSD as the 10% fast ones. 

Second, decide their cooperativity. Delaunay triangulation was used first to identify the nearest neighbor of each fast monomer, and those fast monomers which are in the same tetrahedron are put in the same group. Then, a cut-off inter-monomer distance of 6.5 Å was set to find out if every two fast monomers in the same groups were adjacent. We further judged if every two monomers in the same group were at the same chain or their velocities’ angle was less than 45°. The two monomers needed to meet at least one requirement to prove that they moved cooperatively. Finally, merge groups that have an intersection. 

The algorithm was transferred into codes by Python 3. The mirror coordinates from the coarse-grained MD simulation were used.

## 3. Results

### 3.1. From Neutron Total Scattering to 3D Most Probable All-Atom Structure of PS

The 3D most probable atomic structure of PS is given in Figure 1a. Its temperature dependence Fourier Transforms for PS-d8 (Figure 1b), PS-d5, PS-d3 and their binary blends (Figure S1) were all consistent with the corresponding neutron total scattering curves. Therefore, all frames of MD images represented the 3D most probable all-atom structure of PS. When it was higher than the glass formation temperature, the PS melt was still an ergodic system. One simulation frame represented a spatial average which had the same time average of PS chains; thus, all of the MD images could be regarded as frames of a film, which showed the dynamics of glass formation.

The first peak, *q*_1_, was from the segment–segment interaction (Figure 1b). The segment-segment distance (2 *π*/*q*_1_) decreased from ~10.0 Å at 450 K to ~9.7 Å at 393 K, indicating the formation and shrinkage of the “static cage”. It was a composition of the main chain-main chain (MC-MC), main chain–phenyl (MC-PR) and phenyl-phenyl (PR-PR) interactions (Figure 1c). Because of its amorphous nature, the *q*_1_ peak was broad. The negative contribution from the main chain–phenyl (MC-PR) and phenyl-phenyl (PR-PR) in the *q*_1_ range made the main chain carbon–main chain carbon (MC-MC) distance, 9.5 Å, smaller than 2 *π*/*q*_1_ at 393 K. 

**Figure 2:**
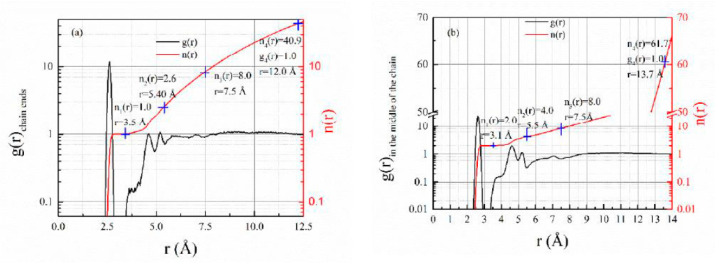
The radial and number distribution functions for chain ends (**a**) and monomers in the middle of chains (**b**) at 393 K. Here, we used the main chain carbon which connected to phenyl ring to represent a monomer.

**Figure 3:**
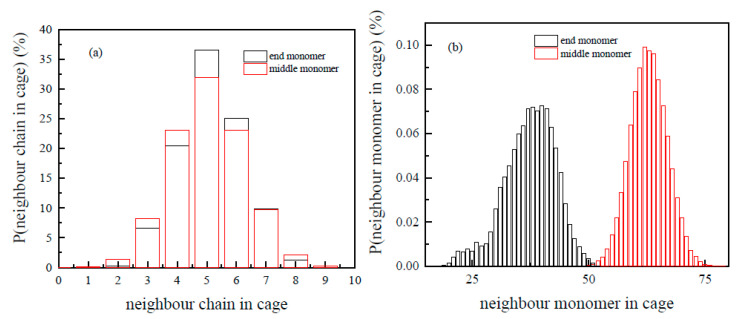
Number distributions of chains (**a**) and monomers (**b**) in “static cage” at 393 *K*. The black bars represent the monomers at the end of the chain, while the red bars represent the monomers in the middle. The cut-off for the monomers at the end of the chain was 12.5 Å, and for the monomers in the middle was 13.7 Å.

“Static cage” structures can be directly seen from the combination of radial and number distributions (Figure 2). There were 47 PS chains inside the 3D box, and each chain had 88 monomers. Therefore, there were only 94 end groups which had more freedom to move; most of the monomers (97.7%) were in the middle of the chains. End groups were only confined at one side (Figure 2a). Their first peak in g(r) proceeded to 3.5 Å, where n(r) continued to show that there was only one neighbor monomer from its own chain; it went to r = 7.5 Å when n(r) = 8; the “static cage” size for the chain end was 12.0 Å (g(r = 12.0) = 1), which, on average, contains about 41.9 monomers. On the other hand, monomers in the middle of the chain were confined at both sides (Figure 2b). Their first peak in g(r) proceeded to 3.1 Å, where n(r) showed there were two neighbors from their own chain; it also continues to r = 7.5 Å when n(r) = 8, after that distances, the monomer number became statistically identical whether the center monomer was at the chain end or in the middle of the chain. The “static cage” size for repeating units in the middle of the chains was 13.7 Å (g(r = 13.7) = 1), which, on average, contains about 62.9 monomers. The shape of g(r) did not change with the decrease in temperature, only the “static cage” shrank. When it came close to glass formation, PS chains would be confined inside the tube from their neighbor. Although the internal motions still existed, they did not affect the viscosity of the melt. There were three things to note here. The first thing was that the number of monomers and chains inside the “static cage” were polydisperse. On average, 1 monomer was confined by 62 neighbor monomers and 5 neighbor chains for the monomer in the middle of chain; 1 monomer was confined by 38 neighbor monomers and 5 neighbor chains for the chain end. Therefore, some of the “static cages” were crowder, and some of them were looser (Figure 3). The second thing was that the “static cage” was different from the “dynamic cage” in the Mode Coupling Theory (MCT). It is generally believed that the size of the “dynamic cage” is significantly smaller than the typical inter-colloid distance [8], and its duration increases quickly with a decreasing temperature [36]. The final thing was that the MC-MC distance could be obtained from the Fourier Transform of Figure 2b, and it was included in Figure 1c (see Figure S2; we used this method to calculate the MC-MC distance in other polymer systems thereafter).

### 3.2. From a 3D Most Probable All-Atom Structure of PS to a Generalized Equation of Excess Free Volume

In a typical polydisperse static cage, PS chains are confined in the tubes formed by their neighbors when glass forms. For the “static cage” with the average number of monomers (${\bar{n}}_{cage}$), the excess free volume (*V_free_*_,*exs*_) is the volume outside of the tubes in the “static cage”. Then, the fractional excess free volume is:

(5) $$\frac{V_{free,exs,Tg}}{V_{total}}=1-\frac{V_{apparent,Tg}}{V_{cage}}=1-\frac{L_{contour}({\bar{n}}_{cage})\pi {\left(\frac{\pi }{q_{MC-MC,Tg}}\right)}^{2}}{V_{cage}}$$

where *V_total_* is the volume of the system; *V_apparent_* is the volume inside the tubes which will not contribute to viscosity; *V_cage_* is the average size of “static cage”; $\frac{\pi }{q_{MC-MC,Tg}}$ is the tube radius when freezing segment motion in the “static cage”, it can be monitored by X-ray or neutron diffraction directly; *L_contour_*(*n*) is the contour length of the polymer chain which has *n* repeating units. 

If the system is close to equilibrium, the average volume of the static cage is the mass of the monomer in the cage divided by its macroscopic density, i.e., $V_{cage}=\frac{m_{cage}}{\rho (T_{g})}=\frac{n_{cage}m_{monomer}}{N_{A}\rho (T_{g})}$, here, *m_monomer_* is the molar mass of monomer, *N_A_* is the Avogadro constant and *ρ*(*T_g_*) is the density at glass formation. Then, Equation (5) becomes:

(6) $$\frac{V_{free,exs,Tg}}{V_{total}}=1-\frac{L_{contour}({\bar{n}}_{cage})\pi {\left(\frac{\pi }{q_{MC-MC,Tg}}\right)}^{2}}{V_{cage}}=1-\frac{L_{contour}({\bar{n}}_{cage})\pi {\left(\frac{\pi }{q_{MC-MC,Tg}}\right)}^{2}}{m_{monomer}}N_{A}\rho (T_{g})$$

Equation (6) partly explains why free volume depends on a different thermal history. *T_g_* here is measured by neutron scattering, i.e., from the temperature-dependent isothermal expansion factor [18], so it could compare with the DSC result. When we changed the temperature or pressure, we mainly changed *V_cage_*. Glass formed when *V_cage_* was so small that the out-of-cage motion was slow enough to avoid being in the measurement range of the instrument. 

For the polymer with a vinyl backbone, *L_contour_*(*n*), the contour length of the polymer chain which had *n* repeating units, could be further simplified:

(7) $$\frac{V_{free,exs,Tg}}{V_{total}}=1-\frac{2L\sin(\theta /2)\pi {\left(\frac{\pi }{q_{MC-MC,Tg}}\right)}^{2}}{m_{monomer}}N_{A}\rho (T_{g})$$

where *L* and *θ* are the bond length and angle, respectively. 

In history, the temperature dependence of free volume was derived from the combination of Doolittle and WLF equations:

(8) $$\frac{V_{free,exs}}{V_{total}}=(\alpha_{L}-\alpha_{G})(T-Tg)+(\frac{V_{free,exs,Tg}}{V_{cage}})$$

where $\alpha =(\frac{1}{v}){\left(\frac{\partial V}{\partial T}\right)}_{P}$, is the thermal expansion factor of a melt or glass.

**Figure 4:**
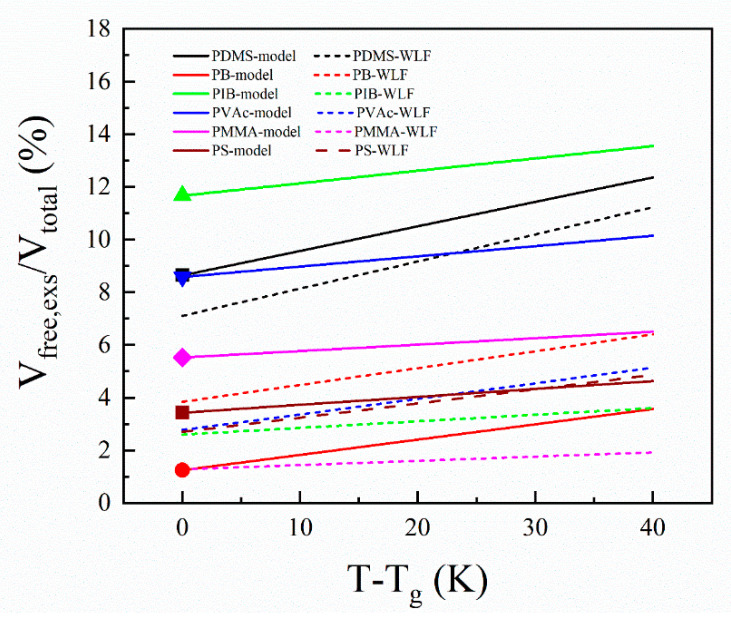
The comparisons of the temperature-dependent fractional excess free volume between the calculation results from Equations (6) or (7) with Equation (8) (solid lines) and WLF results in the literature (dotted lines) for six polymers (the parameters are listed in Table 1 and Table S1).

Because all of the parameters in Equations (6) and (7) can be calculated, we could compare their results with the literature directly. In Figure 4, we analyzed the data for a set of six polymers and compared them with WLF results in the literature (the calculation results are listed in Table 1 and Table S1). The symbols are the fractional free volumes for the six polymers at glass formation, and the solid lines are trends of the free volume over temperatures. The dash lines are the WLF results in the literature. To our knowledge, it’s a good estimation of the WLF free volume [6]. Because there are a few literatures on both computer simulations and scattering experiments with those parameters and their measurement temperatures were away from the glass formation temperature, we could only give limited results and examples now. Prof. Sanditov et al. conducted some researches about the “fluctuation volume” during the glass formation, and they defined “fluctuation volume” as the volume for the delocalization of active atoms [37]. In the future, we will carry out more neutron scattering experiments, combined with an MD simulation to verify our model.

### 3.3. From the Slow Down Dynamics in the Cage to Heterogeneity

How the monomer moves during glass formation is an important question. Because Equation (6) was from the static scattering measurement, it still cannot explain the effect of aging [41]. Whether the monomer moves via free volume or cooperative rearrangement depends on if we can find the exact free volume in the MD images. First, we tried to use probe spheres with different radii to detect every MD image. Here, two traditional ways were used to define the unoccupied volume in the system (see Figure S3), and we tried to find out the connections between the unoccupied volume and free volume. However, as shown in Figure S4, we could not find any free volume-accommodating monomer, whether we used the hard sphere model or soft interaction potentials. Therefore, we had to turn to cooperative rearrangement. To observe dynamic heterogeneity, the simulation time had to be longer than the duration for a monomer to escape out of the cage. Therefore, a coarse-graining model, based on the force fields of the all-atom simulation had to be adopted. The all-atom and CG models were representing the same polystyrene because they had the same interaction potentials. The structures of the CG model at T = 1.0 came from the structures of the all-atom model at 453 K. Although the CG model could only give relative results, it is reliable and widely used to analyze the dynamical characteristics of the system. In the CG model, glass formed when T = 0.4.

**Figure 5:**
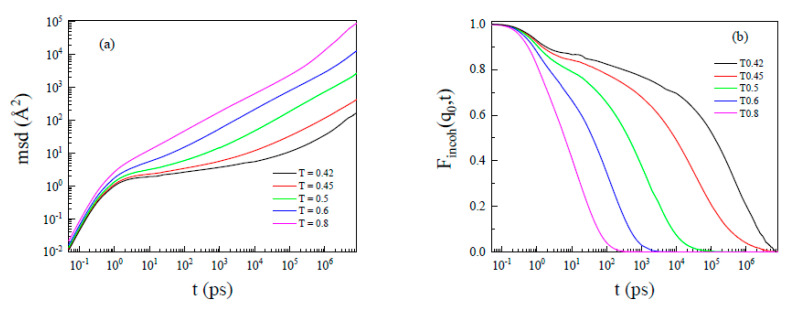
(**a**) Mean square displacements of monomers at different temperatures. (**b**) Incoherent intermediate scattering function of monomers at different temperatures. With the decrease in the temperature, *α*/*β* split happens, and it becomes evident when T = 0.45.

Figure 5a is the mean square displacements (<r_msd_^2^>) of monomers at different temperatures. Leporini and co-workers defined the cage time (β relaxation) in an order of 1 ps [42,43]. Betancourt et al. also simulated the α/β split of a coarse-grained polymer melt and observed an a/β relaxation time of ~1.4 ps [44]. Similar trends were evident here. When it was much higher than glass formation (T = 0.8), <rmsd^2^> continued to increase linearly with time. At T = 0.45, <r_msd_^2^> became a plateau first, as monomers explored its dynamic cage created by its neighbor. Then, at a longer time, <rmsd^2^> grew with cage rearrangement [13]. The development of the α/β split could also be seen from the temperature dependence of the incoherent intermediate scattering function (Figure 5b). The incoherent intermediate scattering function, *F_incoh_* (*q*, *t*), given by:

(9) $$F_{incoh}(q_{0},t)=\frac{1}{N}\sum_{i=1}^{N}b_{j}^{2}<e^{-i\vec{q_{0}}\cdot \vec{R_{j}(0)}}e^{i\vec{q_{0}}\cdot \vec{R_{j}(t)}}>$$

where 2π/*q*_0_ is the main chain–main chain distance. Figure 5b shows a similar tendency of the α/β split. Some researchers showed that heterogeneity develops in the system as it cools down, which causes the violation of the Stokes–Einstein relation [45,46,47,48]. Note that polymer relaxation occurs as a multi-scale hierarchical process involving the cooperative molecular motion. Through the Zwanzig–Mori–Akcasu formalism [49], the solution of the time–position correlation function reduced to an Eigen value problem. However, no one knows how to derive the independent Eigen values here, we only discussed the diffusion motion of monomers thereafter.

To quantify dynamic heterogeneity, the four-point susceptibility, *χ*_4_, was calculated [50]. First, we defined the two-time self-correlation function, $Q_{2}(a,\Delta t)=\frac{1}{Nn}\sum_{i=1}^{Nn}e^{-\frac{\Delta r_{i}^{2}}{2a^{2}}}$, where *a* is a preselected length scale to be probed, $\Delta r_{i}^{2}$ is the mean square displacement of monomer *i* in time Δ*t*, *N* is the number of polymer chains (*N* = 47) and *n* is the degree of polymerization (*n* = 88) in the simulation box. Then, *χ*_4_ is the temporal fluctuations of *Q*_2_,

(10) $$\chi_{4}(a,\Delta t)=Nn(<Q_{2}{\left(a,\Delta t\right)}^{2}>-<Q_{2}(a,\Delta t{)>}^{2})$$

Therefore, *χ*_4_ is linked to the number of monomers participating in a correlated rearrangement.

**Figure 6:**
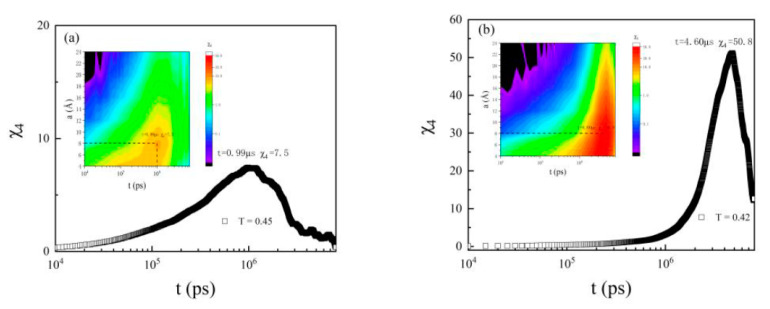
Dynamical susceptibility, χ_4_ for PS monomers at different probe length scales and time scales, at (**a**) T = 0.45 and (**b**) T = 0.42. The maximum dynamic susceptibility was 7.5 at t = 0.99 μs at T = 0.45, and 50.8 at t = 4.6 μs at T = 0.42, respectively. The insets show the trends of χ_4_ with different a and t. The dash lines in the insets were used to indicate the maximum of χ_4_.

The peak lag time was the time over which the dynamics were the most heterogeneous, and the peak height indicated the spatial extent of the heterogeneities. The maximum dynamical susceptibility happened at 0.99 μs at T = 0.45 (Figure 6a), and at 4.60 μs at T = 0.42 (Figure 6b). Thus, during 0.99 μs, CRRs with an average number of 7.4 monomers moved faster at T = 0.45. With the decrease in temperature, both the duration and size of CRRs grew rapidly at T = 0.42.

**Figure 7:**
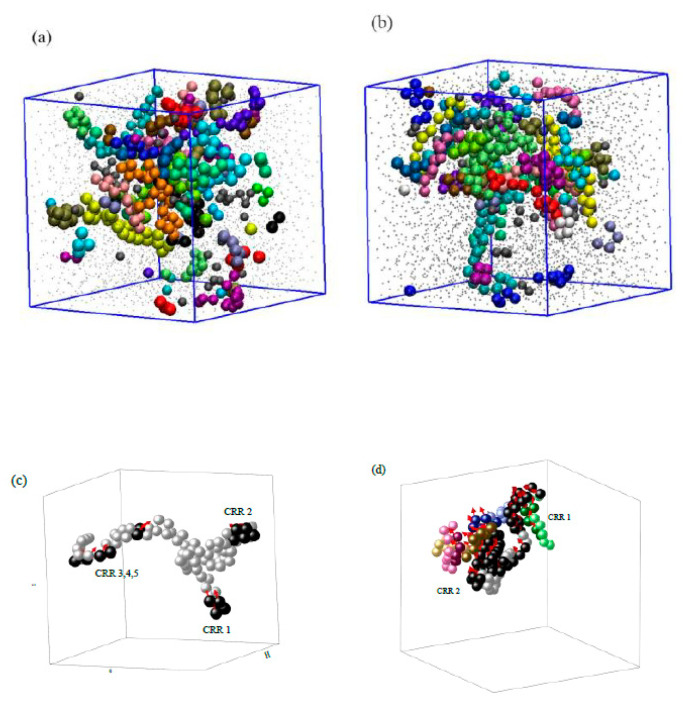
Snapshots of cooperatively rearranging PS monomers. (**a**,**b**) The overall snapshots at T = 0.45 and T = 0.42, respectively. The larger colorful monomers were drawn to scale and represent the 10% fastest ones, different colors represent different chains. The rest of the monomers are shown as small grey dots. Because of periodical boundary conditions, some of the monomers close to the boundary look to be isolated. (**c**,**d**) Typical CRRs at T = 0.45 (CRR1 to CRR5) and T = 0.42 (CRR1 and CRR2), respectively. Different colors mean different chains. Deep colors mean 10% fast monomers, and the light ones mean the rest of the monomers in the same chain. The red arrow shows their moving direction during 0.99 μs at T = 0.45 and 4.60 μs at T = 0.42.

To directly see the size and shape of the dynamic heterogeneity, we followed the methods in the literature [16,17]. When it was close to glass formation, monomers moved and rearranged to out of the cage, and the distinction between the different CRR could only hold over a finite duration. Here, monomers with the 10% largest displacements over a given time (0.99 μs at T = 0.45, and at 4.60 μs at T = 0.42, respectively) were defined as mobile (Figure 7a and Figure 7b). 

All of the CRRs are string-like [51]. At T = 0.45, almost all of the monomers in a string-like CRR belong to the same polymer chain (Figure 7c). More than 36% of the chain ends belong to CRR, indicating their importance to the chain mobility. It can also be seen that most of the fast monomers moved along its backbone; we can, thus, conclude that their interaction was transmitted by the backbone of the Gaussian coil, and most of the fast monomers were crawling along it. It also verifies the tube model in Equations (6) and (7). Zou et al. used X-ray tomography to study the packing of the granular polymer chain, and they found that the suppression of pair-wise contacts between monomers that did not share a bond provided the rigidity [52]. The further decrease in temperature to T = 0.42 led some of the CRRs from different chain to “synchronize” with each other (Figure 7d). The moving direction of strings from different polymer chains “synchronized” over a longer time (4.6 μs), forming larger CRRs. Only 19% of the chain ends belonged to CRRs, showing that their mobility became similar to those in the middle of the chain because of the dynamic slow down.

**Figure 8:**
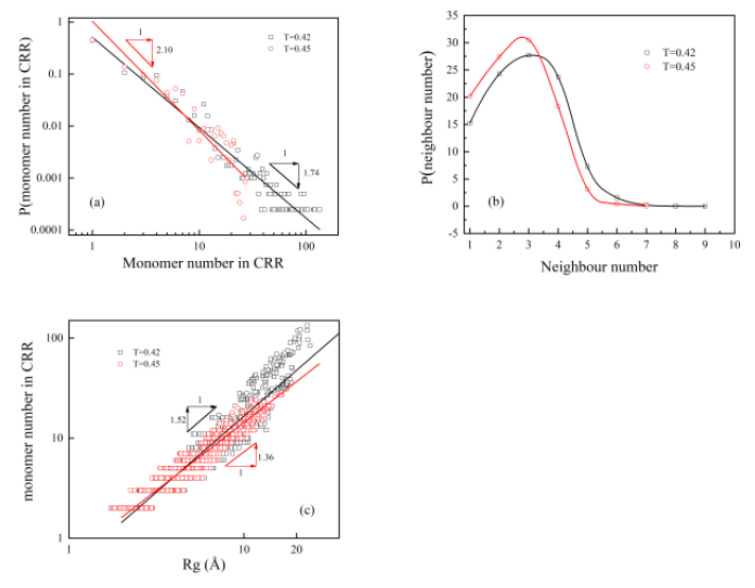
Size and shape of CRRs. (**a**) Number of monomer distribution of sting-like CRRs. The lines are the best fit, with a slope of 2.10 at T = 0.45 and 1.74 at T = 0.42. (**b**) Distribution of the number of neighbors in the CRR. It has an average of 2.5 nearest neighbors at T = 0.45 and 3.0 nearest neighbors at T = 0.42. (c) The radius of gyration dependence of CRR cluster weight. The best fit shows the fractal dimension of the CRR, i.e., 1.36 at T = 0.45 and 1.52 at T = 0.42.

We characterized the nature of the string-like CRRs qualitatively at different temperatures (Figure 8). The size distribution of CRRs was polydisperse. $P(N_{c})\sim N_{c}^{-\upsilon }$ with ν = 2.10 at T = 0.45 and 1.74 at T = 0.42 (Figure 8a). Donati et al. simulated spatial correlations of mobility and immobility in a glass forming a Lennard-Jones liquid [53]. Their result proved that larger CRRs dominate the relaxation process when ν<3 [13,42]. The decrease in ν from T = 0.45 to T = 0.42 proves that larger string-like CRRs with a longer relaxation time dominated the dynamic slow down when it was closer to glass formation. Note that there were only 88 monomers in one polymer chain, and some of the CRRs had more than 100 monomers (Figure 8a). CRRs can, thus, propagate to larger length and time scales with the decrease in aging temperature and the increase in aging time. CRR had an average of 2.5 adjacent neighbors at T = 0.45, and 3.0 neighbors at T = 0.42 (Figure 8b), reflecting its string-like nature. It can further be seen from its fractal dimension (Figure 8c), i.e., df = 1.36 at T = 0.45 and df = 1.52 at T = 0.42. 

### 3.4. Relationship between “Static Cage” and “Dynamic Cage”

Our concept of the “static cage” looks different from the “dynamic cage” in MCT. The former decreases its size, and the latter increases its duration with the decrease in temperature at glass formation. 

The “static cage” and “dynamic cage” can be united as a consequence of a dynamic slow down. With the decrease in thermal fluctuation, both of them formed, and confined the movement of monomers. The “static cage” is a statistical average. We could, thus, calculate the WLF fractional free volume according to the ratio between the occupied volume in the cage and the cage size qualitatively. In fact, because polymer glass is amorphous, the number of monomers inside the “static cage” must be polydisperse. Some of them are crowded, and some of them are loose. Thermal fluctuation balances them dynamically. On the other side, the monomer moves cooperatively to escape from the dynamic cage. Figure S6 counts the monomer number distributions around fast CRR and all monomers in the “static cage” at different temperatures. It indicates that the string-like fast monomers prefer to move inside loose “static cages” during the characteristic lag time. Therefore, the decrease in thermal fluctuation leads to the formation of a polydisperse “static cage”, while the latter “provides” the dynamic path way of CRRs.

## 4. Conclusions

In the present work, we experimentally observed the temperature-dependent atomic structure of a PS melt, and demonstrated whether PS moves via free volume or cooperative rearrangement during glass formation. A simple equation was posed to calculate the fractional excess free volume in the polymer system. The key parameter in the equation, $\frac{\pi }{q_{MC-MC,Tg}}$, can be derived by a combination of scattering experiments and MD simulations. Additionally, its calculation result can be verified with the measurement result of the WLF equation directly. However, this excess free volume is just a statistical average, the free volume that can accommodate the monomer can never be discovered in any frame of its real space image. Therefore, free volume is a statistical average, and monomers, thus, have to move cooperatively to escape out of the cage. String-like cooperate rearranged regions develop their lengths and duration when it is close to glass formation. 

We believe that both free volume and dynamic heterogeneity are consequences of the dynamic slow down. The former is a statistical average. It leads to the formation of the polydisperse “static cage”. Some of the “static cages” are crowded, and some of them are loose. The polydisperse “static cage” defines the way that CRRs move. The dynamic balance between the crowded and loose “static cage” may reflect the amplitude of dynamic heterogeneity. Our work provides a universal microscopic picture of how a polymer moves during glass formation.

## Figures and Tables

**Table 1:** The temperature-dependent fractional excess free volume when it is close to glass formation. Calculation results according to both our model (Equations (6)–(8)) and WLF equation in the literature (if *α_G_* could not be found in the literature, only *α_L_* was used).

Polymer	T_g_ (K)	Calculation Results According to Equations (6)–(8)		WLF Results from Literature ^a^	
		Fractional Excess Free volume at T_g_ (%)	*α_L_*-*α_G_* (×10^−4^ K^−1^)	Fractional Excess Free volume at T_g_ (%)	Slope (×10^−4^)
PDMS	150	8.6	9.3 ^b^	7.1	10.3
PB	172	1.2	5.8 ^c^	3.8	6.4
PIB	205	11.7	4.7 ^d^	2.6	2.5
PVAc	305	8.6	3.9 ^b^	2.8	5.9
PMMA	381	5.5	2.5 ^b^	1.3	1.6
PS-h8	373	1.5	3.0	2.7	5.4

^a^ See Ref [13]. ^b^ See Ref [38]. ^c^ See Ref [39]. ^d^ See Ref [40].

## Data Availability

The data that supports the findings of this study are available within the article and its supplementary material.

## Supplementary Materials

The following are available online at https://www.mdpi.com/article/10.3390/polym13183042/s1, Figure S1: Neutron total scattering and MD simulations; Figure S2: Main chain-main chain distances in reciprocal space from both the decomposition of q1 peak and the Fourier Transform of g(r) in Figure 2; Table S1: Parameters to calculate Equation (5) for six polymers; Figure S3: Real space image of the unoccupied volume at 393 K, 423 K and 453 K; Figure S4: The temperature dependence unoccupied volumes with different probe sizes; Figure S5: Snapshots of another way to color the fast monomers in CRRs; Figure S6: Monomer number distribution in “static cage” around all monomers and fast 10% ones at different temperatures.
