# Supplementary material for: The Relationship between Free Volume and Cooperative Rearrangement: From the Temperature-Dependent Neutron Total Scattering Experiment of Polystyrene
Source: Polymers (Basel). 2021 Sep 9;13(18):3042. doi: 10.3390/polym13183042 (PMC8470135; doi:10.3390/polym13183042)
Supplement: Supplementary file 1 [file polymers-13-03042-s001.zip › polymers-1362914-supplementary.pdf]

Supplemental Material for:

The Relationship between Free Volume and Cooperative Rearrangement: from the Temperature-Dependent Neutron Total Scattering Experiment of Polystyrene

Zehua Han [1,2,3,a)] Guisheng Jiao [4,5,a)] Changli Ma,[1,2] Taisen Zuo,[1,2,*] Charles C. Han,[6] and He Cheng,[1,2,*]

[1] Spallation Neutron Source Science Center (SNSSC), Dongguan 523808, China; ZH. Han: hanzh@ihep.ac.cn

[2] Institute of High Energy Physics (IHEP), Chinese Academy of Science (CAS), Beijing 100049, China; CL Ma: machangli@ihep.ac.cn

[3] University of Chinese Academy of Sciences, Beijing 100049, China

[4] Shenzhen Key Laboratory of Polymer Science and Technology, Guangdong Research Center for Interfacial Engineering of Functional Materials, College of Materials Science and Engineering, Shenzhen University, Shenzhen 518055, China； GS. Jiao: jiaogs@szu.edu.cn

[5] College of Physics and Optoelectronic Engineering, Shenzhen University, Shenzhen 518060, China

[6] Institute for Advanced Study, Shenzhen University, Shenzhen, 508060, China； C. C. Han: han.polymer02@yahoo.com

* Correspondence: TS. Zuo, Zuots@ihep.ac.cn; H. Cheng, chenghe@ihep.ac.cn; Tel.: Tel: +86-769-8915-6445; Fax: +86-769-8915-6441

[a)]These authors contribute equally in the work.

[b)]Authors to whom correspondence should be adressed:

Taisen Zuo, E-mail: Zuots@ihep.ac.cn; He Cheng, E-mail: chenghe@ihep.ac.cn,

Tel: +86-769-8915-6445; Fax: +86-769-8915-6441.

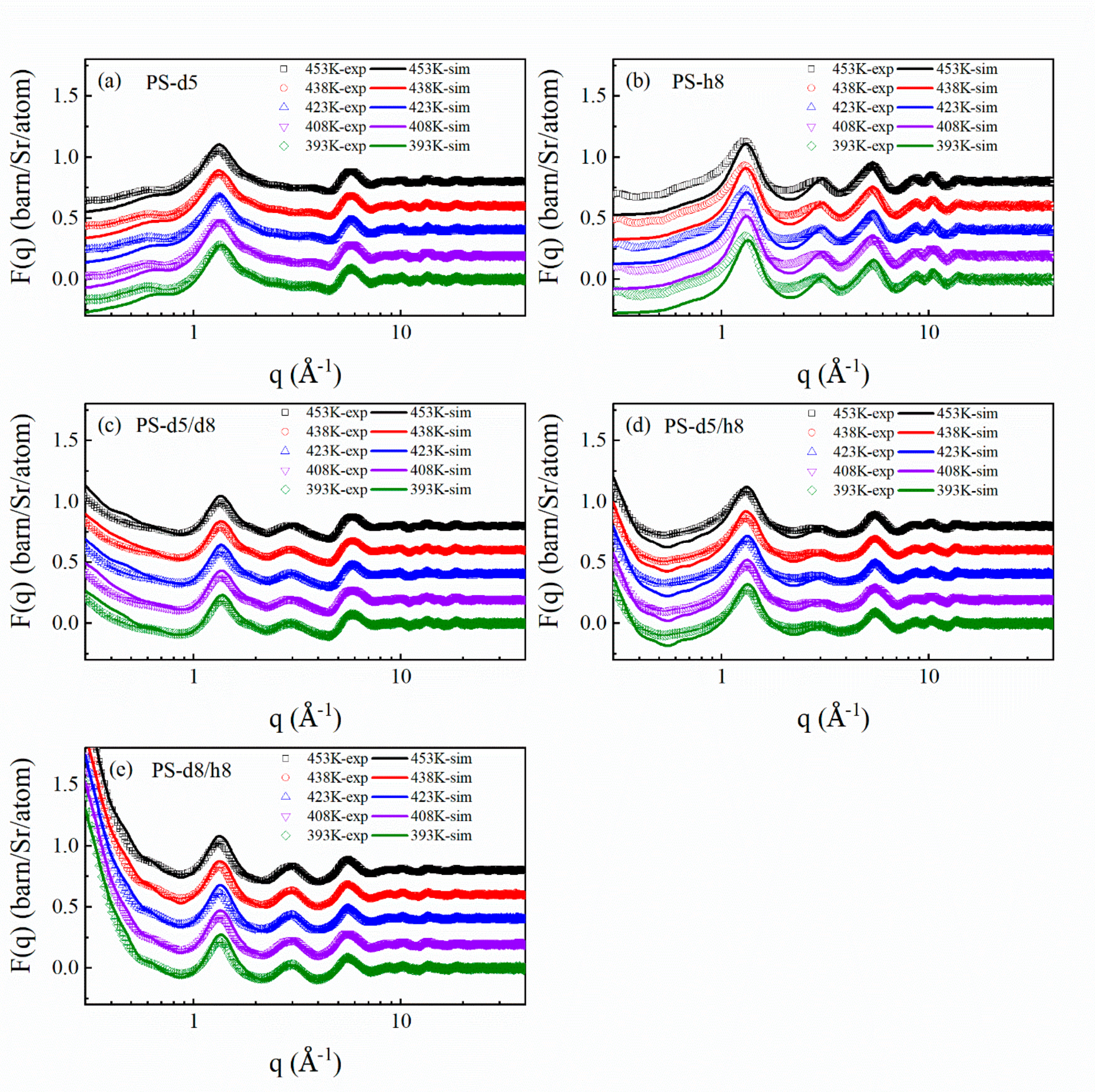

Figure S1. Neutron total scattering and MD simulations. The neutron total scattering profiles of PS-h8, PS-d5, PS-d5/d8 and their binary mixtures with PS-d8 at different temperatures, e. g., 453*K*, 438*K*, 423*K*, 408*K*, and 393*K*. The symbols are data of experiments and the lines are from the Fourier Transforms from the correlation function of the corresponding MD simulations.

The 3d most-probable atomic structure of PS is given in Figure 1(a). Its temperature dependence Fourier Transforms for PS-d8 (Figure 1(b)), PS-d5, PS-d3 and their binary blends (Figure S1), are all consistent with the corresponding neutron total scattering curves. Therefore, all frames of MD images represent the 3d most-probable all atom structure of PS.

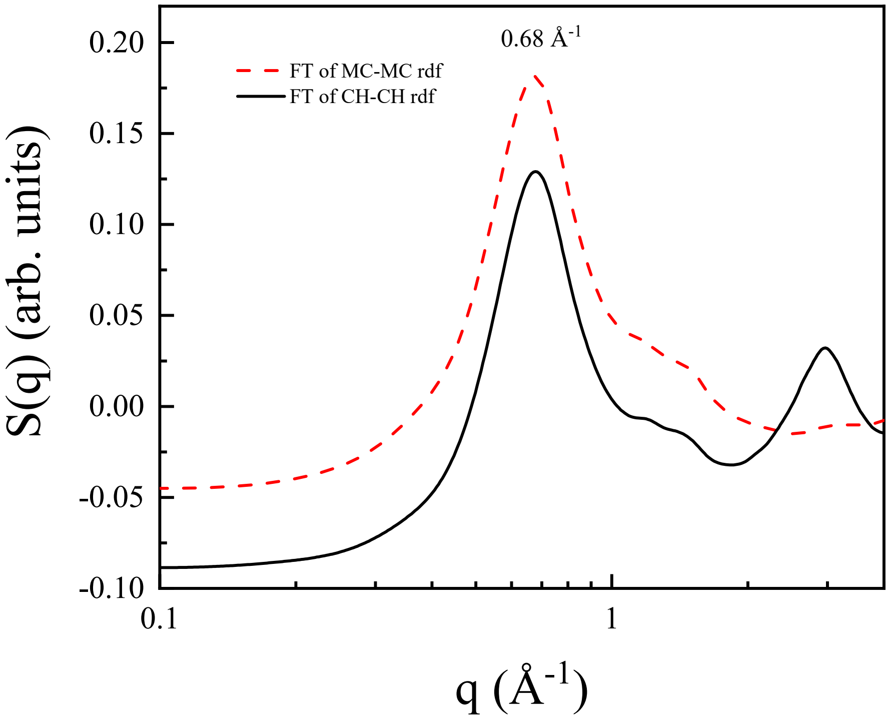

Figure S2. Main chain-main chain distances in reciprocal space from both the decomposition of q_1 peak (black line) and the Fourier Transform of g(r) in Fig. 2 (red dot).

Figure S2 proves that MC-MC distance can be gotten from the Fourier Transform of Figure 2(b), and it is consisted with Figure 1(c). It means that the average main chain – main chain distance can be calculated from simulation result in literature. We will use this method to calculate MC-MC distance in other polymer systems thereafter.

From 3d most probable all-atom structure of PS to a generalized equation of excess free volume.

To calculate the parameters in Table 1, some basic data are needed. They are shown in Table S1.

Table S1. Parameters to calculate eq 5 for six polymers

Polymer	Contour length per monomer ($\AA$)[a]	Radius ($\AA$)[b]	Density at glass point (g/cm^3)[c]
**PDMS**	2.65	3.50[d]	1.10[e]
**PB**	5.03	2.30[f]	1.15[e]
**PIB**	2.60	3.20[g]	0.96[g]
**PVAc**	2.53	3.60[h]	1.19[e]
**PMMA**	2.53	4.30[i]	1.07[e]
**PS**	2.45	4.60	1.02

[a] From the OPLS-AA force field.

[b] Calculated according to the results of MD simulations or scattering experiments in the references. We did Fourier Transform of rdf of the main chain atom – main chain atom for polymers with MD simulations. For polymers with only scattering experiments, we use the first peak to calculate the distance.

[c] Calculated according to eq. 6 or 7.

[d]See Ref. [1]
[e]See Ref. [2]
[f]See Ref. [3]
[g]See Ref. [4]
[h]See Ref. [5]
[i]See Ref. [6]

From the slow-down dynamics in cage to heterogeneity.

When we are using data from Bondi to define Van der Waals radius for every atom, each atom can be treated as a hard sphere. The space within the Van der Waals radius is the occupied volume. The rest part is the unoccupied volume. Generally, there are two ways to calculate the unoccupied volume in literatures.

The first one is used by Gromacs software. It randomly puts more than thousands of points with almost zero radius in the image to test if the points are beyond all the Van der Waals spheres. It can finally get a ratio of points in unoccupied volume. However, this way can only give the ratio of the unoccupied volume, and cannot get their exact positions.

The second one is extensively used by many groups, especially in gas storage and gas transportation fields. We can divide the whole image into multi cells with equal size along three axes. We can thus get a lattice, in which each cell presents a mini volume. Then we can test if the cell is beyond all the Van der Waals balls. Finally, we can store the coordinates of all the cells which is not occupied by atoms, and get a direct image of the unoccupied volume.

We used both methods, so we can get a direct image of the unoccupied volume (Figure S3), and we can also prove that the results from both ways are almost the same.

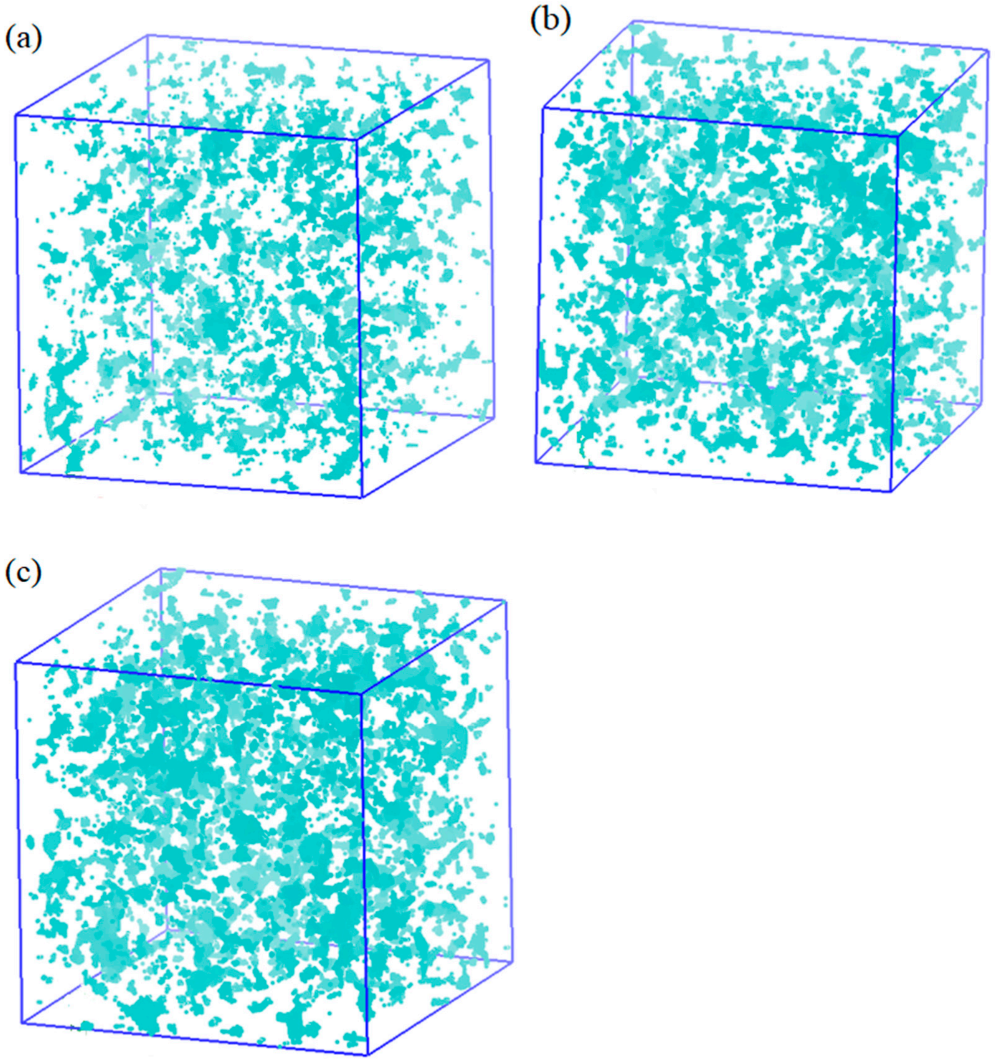

Figure S3. Real space image of the unoccupied volume at (a) 393K, (b) 423K and (c) 453K. The green area represents the volume which is not occupied by the Van der Waals volume of the atoms.

Just as mentioned in Figure S3, the unoccupied volume can be calculated from the direct image of the MD results. Thus, we can get the temperature dependence of the unoccupied volume with different probe sizes. If the fluctuations at different temperatures are considered, the Van der Waals volume can be calculated using a soft sphere model. We show the results in Fig S4. No connections can be found between the unoccupied volume and the results of WLF.

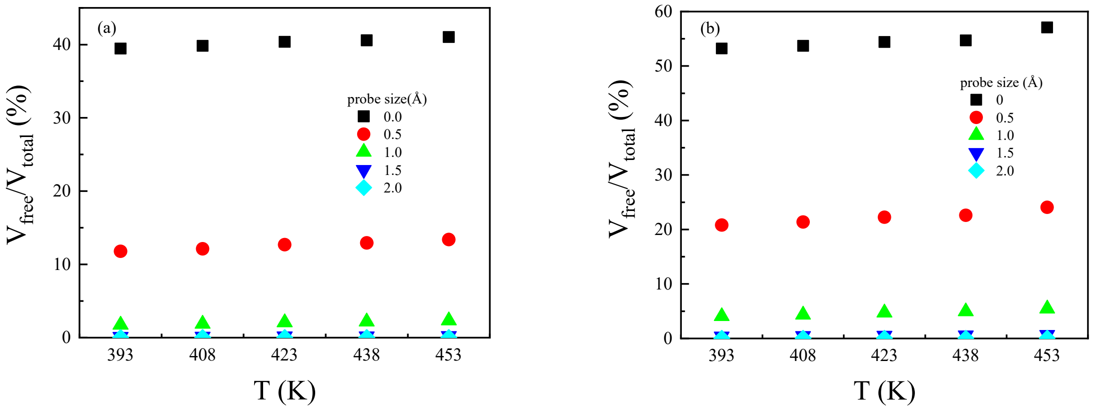

Figure S4. The temperature dependence unoccupied volumes with different probe sizes. (a) Van de Waals radii are used to define the boundary of different atoms; (b) Thermal fluctuation is taken into account. There is no space in the system that can contain even one monomer.

Figure S5 shows another way to color the fast monomers. The monomers are colored by the CRR they belong to. Compared to Figure 7(a) and Figure 7(b), it's obvious that at T = 0.42, the size of CRR is larger, and one big CRR generally contains atoms from more than one chain.

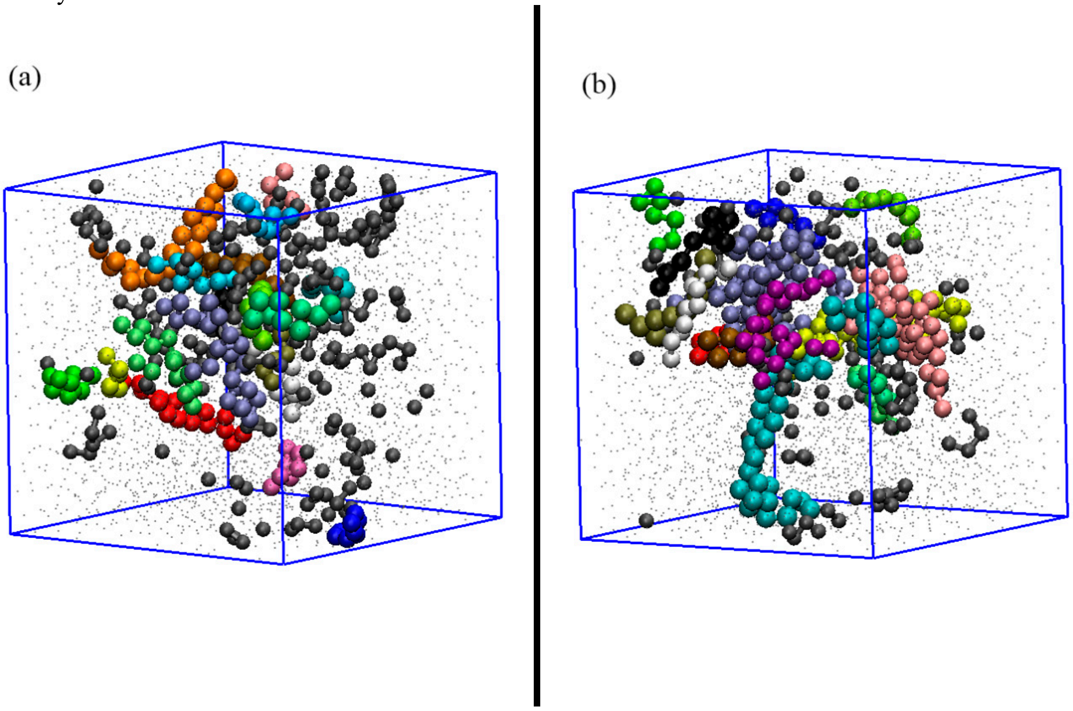

Figure S5. Snapshots of CRRs at (a) T=0.45 and (b) T=0.42, respectively. The larger colorful monomers are drawn to scale and represent the 10% fastest ones, different color represents different CRRs. The rest of the monomers are shown as small grey dots. Because of periodical boundary conditions, some of the monomers close to the boundary look to be isolated.

Relationship between "static cage" and "dynamic cage"

The local environment for 10% fast monomers. It's easy to see, at both temperatures, there are less neighbors around the fast monomers, which means the local environment for fast monomers is loose.

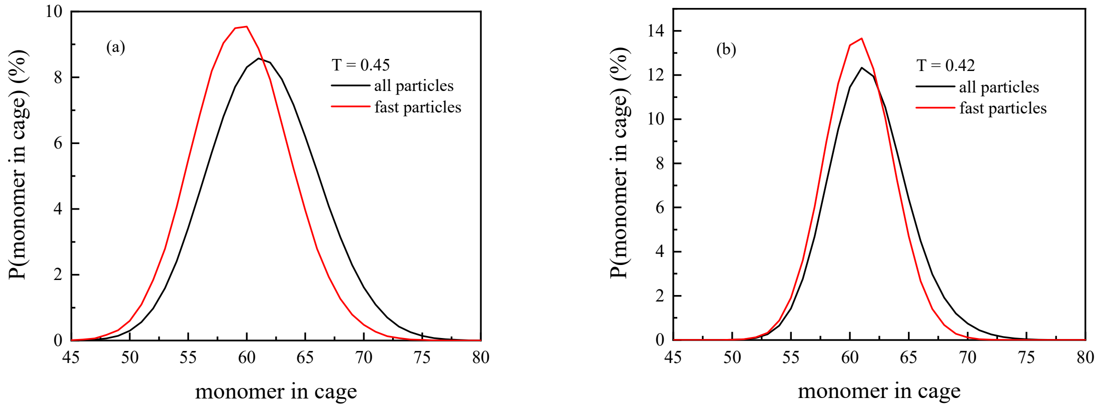

Figure S6. Monomer number distribution in "static cage" around all monomers (blackline) and fast 10% CRR monomers (red line) at different temperatures. (a) T=0.45, (b) T=0.42. At both temperatures, the neighbors around fast monomers are always less.

References

1. H. Sun, D. Rigby. *Spectrochimica Acta Part A: Molecular and Biomolecular Spectroscopy.* **1997**, *53,* 1301.
2. R. A. Orwoll, in Physical properties of polymers handbook. *Springer*, **2007**, 93-101.
3. A. Narros *et al*., *Macromolecules.* **2005**, *38,* 9847.
4. R. H. Boyd, P. V. K. Pant, *Macromolecules* **1991**, *24,* 6325.
5. M. Tyagi, A. Arbe, F. Alvarez, J. Colmenero, M. A. González, *J. Chem. Phys* **2008**, *129,* 224903.
6. A. C. Genix *et al*., Macromolecules **2006**, *39,* 3947.
